# A263 MODULATION OF THE GUT MICROBIOTA TO PREVENT LOCAL ANASTOMOTIC TUMORS AND DISTANT METASTASIS IN COLORECTAL CANCER SURGERY

**DOI:** 10.1093/jcag/gwac036.263

**Published:** 2023-03-07

**Authors:** R Hajjar, A S Ajayi, M Oliero, G Fragoso, A A Alaoui, T Cuisiniere, C Gerkins, A Calvé, H Vennin Rendos, C Richard, M M Santos

**Affiliations:** 1 Université de Montréal; 2 CRCHUM; 3 Surgery, CHUM; 4 Axe cancer, CRCHUM, Montreal, Canada

## Abstract

**Background:**

Colorectal cancer (CRC) recurrence is a leading cause of mortality worldwide. It has been suggested that poor anastomotic healing and leakage (AL) after surgery allows cancer cells to implant at the anastomotic site thereby increasing the risk of local cancer recurrence and metastatic spread in the peritoneum and to extraintestinal organs. We have previously showed that inulin, a well-known prebiotic, improves anastomotic healing and strengthens the gut barrier.

**Purpose:**

The objective of the this study was to investigate the relationship between the promotion of postoperative intestinal healing using inulin and local and distant cancer recurrence.

**Method:**

A 10-years' retrospective review of AL and non-AL cases after CRC surgery was performed in our institution. The effect of dietary supplementation with inulin on the occurrence of local anastomotic tumors was assessed in a mouse model inoculated with tumor cells directly in the gut lumen after colonic surgery. We also investigated in mice whether inulin may prevent metastatic spread and growth of tumor cells in the liver by transplanting CRC cells surgically into the spleen.

**Result(s):**

Patients experiencing AL displayed significantly lower overall survival and more cancer recurrence and progression compared to non-AL patients. Poor anastomotic healing in mice led to larger anastomotic tumors and peritoneal cancer dissemination. Inulin supplementation significantly inhibited local tumor implantation at the anastomotic site, and metastatic spread to the peritoneum and liver. Inulin increased the production of beneficial short-chain fatty acids, reinforced the gut barrier function and alleviated systemic inflammation in mice.

**Conclusion(s):**

Anastomotic leak was associated with worse oncological outcomes in patients and mice. Inulin was shown to reinforce the gut barrier, decrease the implantation of cancer cells at the anastomosis, and to prevent tumor dissemination and progression of liver metastasis. This paves the way toward future clinical trials in which such supplementation may be used to promote better oncological outcomes.

**Please acknowledge all funding agencies by checking the applicable boxes below:**

CIHR, Other

**Please indicate your source of funding;:**

CRS, FRQS

**Disclosure of Interest:**

None Declared

